# Real‐World Outcomes of Brigatinib Compared to Alectinib as a Second‐Line Therapy After Crizotinib in Advanced Anaplastic Lymphoma Kinase Positive Non‐Small Cell Lung Cancer Patients

**DOI:** 10.1111/1759-7714.70175

**Published:** 2025-10-10

**Authors:** Min Jee Kim, Hyun Seok Kwak, Eun Nim Koh, Cheol‐Kyu Park, Young‐Chul Kim, In‐Jae Oh, Seung Joon Kim, Jun Hyeok Lim, Jeong‐Seon Ryu, Chang Min Choi

**Affiliations:** ^1^ Department of Pulmonary and Critical Care Medicine Asan Medical Center, University of Ulsan College of Medicine Seoul Republic of Korea; ^2^ Department of Internal Medicine Chonnam National University Medical School Hwasun Republic of Korea; ^3^ Division of Pulmonology, Department of Internal Medicine Seoul St. Mary's Hospital, College of Medicine, The Catholic University of Korea Seoul Republic of Korea; ^4^ Division of Pulmonology, Department of Internal Medicine Inha University Hospital, Inha University College of Medicine Incheon Republic of Korea

**Keywords:** Alectinib, ALK‐positive non‐small cell lung cacner, Brigatinib, second‐line treatment

## Abstract

**Background:**

Second‐generation anaplastic lymphoma kinase (ALK) inhibitors, including alectinib and brigatinib, are widely used in patients with ALK‐positive non‐small cell lung cancer (NSCLC) who develop resistance or progress on crizotinib. However, real‐world data comparing their efficacy and safety remain limited. This multicenter, prospective cohort study compared the clinical outcomes of alectinib and brigatinib in this setting.

**Methods:**

Patients with stage IV ALK‐positive NSCLC who progressed on crizotinib were enrolled and treated with either brigatinib or alectinib. The primary endpoint was the progression‐free survival (PFS) rate.

**Results:**

Sixty patients were included (brigatinib, *n* = 34; alectinib, *n* = 26). Median follow‐up durations were 26.5 and 30.0 months. Disease progression or death occurred in 50.0% (brigatinib) and 46.2% (alectinib), respectively. The 3‐year PFS was 51.5% (brigatinib) vs. 62.1% (alectinib), with no significant difference at 5 years (40.0% vs. 42.5%; *p* = 0.260). Overall response rates were similar (58.8% vs. 46.2%; *p* = 0.475). However, intracranial outcomes appeared more favorable with alectinib: the 3‐year intracranial PFS was 70.5% vs. 31.7% (*p* = 0.023), and intracranial ORR was 94.4% vs. 64.3% (*p* = 0.028). More patients in the brigatinib group had prior whole‐brain radiotherapy (21.4% vs. 5.6%), while radiosurgery was more frequent in the alectinib group (55.6% vs. 35.7%). Treatment discontinuation rates due to adverse events were comparable between the two groups.

**Conclusions:**

In crizotinib‐refractory ALK‐positive NSCLC, systemic efficacy was not significantly different between brigatinib and alectinib; however, alectinib was associated with more favorable intracranial PFS and ORR, which may be partly explained by differences in prior brain‐directed local treatments.

## Graphical abstract

In this prospective multicenter study of crizotinib‐refractory ALK‐positive NSCLC, the systemic efficacy of brigatinib and alectinib was not significantly different. Differences in intracranial progression‐free survival and response rates were observed, with outcomes appearing more favorable for alectinib.

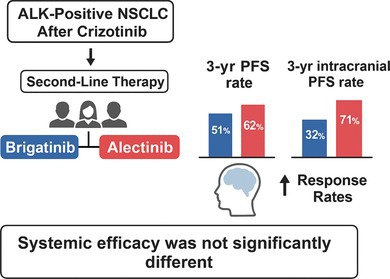

## 1 Introduction

Lung cancer is the leading cause of death worldwide [1]. Despite notable advancements in treatment over the past decades, lung cancer remains highly fatal, with more than half of patients dying within the first year of diagnosis and a five‐year survival rate of 18% [2]. Non‐small cell lung cancer (NSCLC) accounts for 80%–85% of all cases of lung cancer, adenocarcinoma (ADC) being one of the most common histological subtypes [3]. Approximately 40% of patients with NSCLC are diagnosed at a metastatic or locally advanced stage, associated with a poor prognosis. Traditionally, platinum‐based chemotherapy has historically been the first‐line therapy of choice for advanced NSCLC among patients without driver mutations. Over the past decades, various driver mutations and their corresponding targeted agents have been developed, emerging as effective treatment options for advanced NSCLC [4, 5].

Anaplastic lymphoma kinase (ALK) gene rearrangement is activated by the EML4‐ALK fusion gene that encodes a cytoplasmic chimeric protein with constitutive kinase activity [6] and found in 3%–5% of advanced NSCLC patients [7, 8]. Crizotinib, a first‐generation ALK inhibitor, showed clinical efficacy in the progression‐free survival (PFS) and objective response rate (ORR) in a previous 3‐phase clinical trial [9] and was approved by the US Food and Drug Administration for the treatment of patients with advanced NSCLC harboring ALK gene translocation [10].

However, despite its efficacy, resistance to crizotinib commonly develops, and disease progression in the central nervous system (CNS) is frequently observed, likely due to the poor brain penetration [11]. Potential mechanisms of resistance to ALK inhibitors include secondary ALK mutations and bypass signaling through activation of other receptor tyrosine kinases [12]. To address these limitations, second‐generation ALK inhibitors, such as ceritinib, alectinib, and brigatinib, have been developed, offering improved CNS penetration and more potent ALK inhibition [13, 14].

Brigatinib is a secondary generation ALK inhibitor effective against a variety of ALK mutations and ROS rearrangements [15, 16, 17]. It is the only ALK inhibitor that demonstrates efficacy in cell lines with mutations in the epidermal growth factor receptor gene [15, 16, 17]. In a randomized, multicenter phase II trial, a 180 mg dose of brigatinib demonstrated an ORR of 45% and a median PFS of 12.9 months in crizotinib‐refractory ALK‐positive NSCLC patients [18]. Recently, Yang et al. reported the clinical efficacy of brigatinib compared to alectinib in a randomized, multicenter, head‐to‐head phase III trial (ALTA‐3), demonstrating comparable PFS and safety profiles [19].

However, data regarding the effectiveness and clinical outcomes of ALK inhibitors in real‐world clinical practice, rather than in highly controlled trial settings, remain limited. Furthermore, there is a lack of comparative data on the efficacy of ALK inhibitors in patients with crizotinib‐refractory NSCLC [20]. Therefore, we aimed to explore whether brigatinib and alectinib would demonstrate similar systemic efficacy in the real‐world setting after crizotinib failure, and to compare their clinical outcomes in patients with ALK‐positive NSCLC.

## 2 Methods

### 2.1 Study Design and Patients

This study was a prospective, multi‐center cohort study performed at 4 medical centers in South Korea between April 2021 and January 2023 designed to compare and analyze the real‐world clinical outcomes of brigatinib and alectinib. Eligible patients were ≥ 18 years old and had a diagnosis of ALK positive NSCLC, confirmed by tissue biopsy or cytology, with locally advanced or metastatic disease. Patients were required to have experienced disease progression after receiving crizotinib as first‐line treatment for at least 84 days. Definitive concurrent chemoradiotherapy, neo‐adjuvant or adjuvant chemotherapy administered prior to crizotinib treatment was permitted. The exclusion criteria included patients treated with ALK inhibitors other than alectinib or brigatinib, or who received chemotherapy following disease progression on prior crizotinib. Patients who discontinued first‐line crizotinib treatment for reasons other than disease progression were also excluded. All consecutive patients who met the eligibility criteria during the study period were prospectively enrolled, and no patients were excluded after screening.

Treatment decisions were made at the physician's discretion, considering patient characteristics such as baseline comorbidities and performance status, the treating physician's prior clinical experience and the known toxicity profiles of the agents. Brigatinib was administered at 180 mg once daily following a 7‐day lead‐in at 90 mg once daily, and alectinib was administered at 600 mg twice daily. Dose reductions or treatment discontinuations were made at the physician's discretion according to clinical benefit and tolerability.

The study was approved by the Institutional Review Board of Asan Medical Center (IRB No. 2020‐1577) as well as by the IRBs of all participating centers. In accordance with local regulations, each institution granted approval for the use of patient data for the purposes of this study. The study was registered at the Clinical Research Information Service (CRIS; registry number, KCT0005952, date of first registration: 22/02/2021). Written informed consent was obtained from all participants.

### 2.2 Clinical Data Collection

Demographic data including age, sex, Eastern Cooperative Oncology Group performance score (ECOG PS), and smoking history, and cancer‐related information including histologic type of lung cancer, clinical stage, method of ALK test, intracranial involvement, and previous anticancer treatment history were collected from medical records. All baseline demographic and clinical characteristics were assessed at the time of initiation of the index post‐crizotinib therapy (alectinib or brigatinib).

### 2.3 Outcomes

The primary endpoint of this study was PFS rate. The secondary endpoint included intracranial PFS rate, ORR, intracranial ORR, disease control rate (DCR), intracranial DCR, safety, and tolerability. We evaluated PFS from the first day of second‐line ALK inhibitor treatment to disease progression or death. Intracranial PFS was defined as the time from the first day of second‐line ALK inhibitor treatment until the date of intracranial progression or death from any cause in the absence of known intracranial progression. The ORR was defined as the percentage of patients who had at least one complete response (CR) or partial response (PR) prior to any evidence of progression. DCR was defined as the proportion of patients achieving a best response of CR, PR, or stable disease (SD). The overall survival (OS) was defined as the time from the initiation of each treatment to death from any cause. Patients who were alive at the analysis cutoff date were censored at that point. Researchers evaluated the status of disease progression based on the Response Evaluation Criterion in Solid Tumors, ver. 1.1.

### 2.4 Statistical Analysis

All data are expressed as mean ± standard deviation or median (interquartile ranges) for continuous variables and percentages for categorical variables. The Student's *t*‐test was used for continuous data, and Pearson's Chi‐square or Fisher's exact tests were used for categorical data. We used the Kaplan–Meier estimates, and the log‐rank test was used for survival analysis to estimate the median time to event. *p*‐Values < 0.05 were considered statistically significant. Statistical analysis was performed using the R software version 4.2.1 (the R Foundation, Vienna, Austria).

## 3 Results

### 3.1 Baseline Characteristics

Between April 2021 and January 2023, a total of 60 eligible patients with ALK‐positive NSCLC who were refractory to first‐line crizotinib treatment were included in the study. The median follow‐up duration was 26.5 months (95% confidence interval [CI], 16–35 months) in the brigatinib group and 30.0 months (95% CI, 24–47 months) in the alectinib group. Baseline characteristics of the study population are summarized in Table 1. The mean age was 57.2 years, and 53.3% of the patients were female. Most patients were never‐smokers (60.0%), followed by ex‐smokers (23.3%) and current smokers (16.7%). The majority had an ECOG PS of 1 (91.4%), and all patients had adenocarcinoma.

**TABLE 1:** Baseline characteristics of the study population.

Characteristics	Total	Brigatinib	Alectinib	*p*
Number of patients	60	34	26	
Age, years	57.2 ± 10.8	57.4 ± 9.7	56.8 ± 12.3	0.824
Sex				0.848
Male	28 (46.7)	15 (44.1)	13 (50.0)	
Female	32 (53.3)	19 (55.9)	13 (50.0)	
Smoking status				0.124
Never smoker	36 (60.0)	21 (61.8)	15 (57.7)	
Ex‐smoker	14 (23.3)	10 (29.4)	4 (15.4)	
Current smoker	10 (16.7)	3 (8.8)	7 (26.9)	
ECOG PS				0.932
0	3 (5.2)	2 (5.9)	1 (4.2)	
1	53 (91.4)	31 (91.2)	22 (91.7)	
2	2 (3.4)	1 (2.9)	1 (4.2)	
Disease stage				0.241
III	6 (10.0)	3 (8.8)	3 (11.5)	
IIIA	2 (3.3)	2 (5.9)	0 (0)	
IIIB	2 (3.3)	0 (0)	2 (7.7)	
IIIC	2 (3.3)	1 (2.9)	1 (3.8)	
Recurrence	2 (3.3)	1 (2.9)	1 (3.8)	
IV	54 (90.0)	31 (91.2)	23 (88.5)	
Recurrence	12 (20.0)	5 (14.7)	7 (27.0)	
Number of metastatic sites	1 (1–5)	1 (1–5)	1 (1–4)	
Histologic type				0.187
Adenocarcinoma	60 (100)	34 (100)	26 (100)	
Baseline brain metastasis				
Yes	32 (53.3)	14 (41.2)	18 (69.2)	0.058
Prior surgery	3 (9.4)	0 (0)	3 (11.5)	
Prior radiosurgery	15 (46.9)	5 (35.7)	10 (55.6)	
Prior WBRT	4 (12.5)	3 (21.4)	1 (5.6)	
No prior local treatment	14 (43.8)	7 (50.0)	7 (38.9)	
No	28 (46.7)	20 (58.8)	8 (30.8)	
ALK test method				0.012
FISH	25 (41.7)	17 (50.0)	8 (30.8)	
Immunohistochemistry	19 (31.7)	13 (38.2)	6 (23.1)	
Both	16 (26.7)	4 (11.8)	12 (46.2)	
Previous chemotherapy				0.428
No prior chemotherapy	51 (85.0)	30 (88.2)	21 (80.8)	
Neoadjuvant chemotherapy	2 (3.3)	1 (2.9)	1 (3.8)	
Adjuvant chemotherapy	5 (8.3)	3 (8.8)	2 (7.7)	
Definite chemoradiotherapy	2 (3.3)	0 (0)	2 (7.7)	
Duration of prior crizotinib treatment, months	19.6 ± 20.5	17.3 ± 15.2	22.7 ± 25.8	0.346

*Note:* Data are presented as mean ± standard deviation, or number (%).

Abbreviations: ALK, anaplastic lymphoma kinase; ECOG PS, Eastern Cooperative Oncology Group performance status; FISH, fluorescence in situ hybridization; WBRT, whole brain radiotherapy.

At baseline, most patients had stage IV disease (90.0%), while stage III accounted for 10.0% (IIIA 3.3%, IIIB 3.3%, IIIC 3.3%). Recurrent disease at enrollment was observed in 33.3% of patients with stage III and 12 patients (22.2%) with stage IV (brigatinib 5/31 [14.7%] vs. alectinib 7/23 [27.0%]). The distribution of stage III and IV disease was similar across treatment groups, although the proportion of patients with recurrent disease was numerically higher in the alectinib group than in the brigatinib group. The baseline metastatic burden, defined as the number of metastatic organs, had a median of 1 (range, 1–5) overall and was comparable between groups (brigatinib 1 [1–5] vs. alectinib 1 [1–4]).

Intracranial metastases were present in 32 patients (53.3%) at the initiation of second‐line ALK inhibitor treatment, with 14 patients (41.2%) in the brigatinib group and 18 patients (69.2%) in the alectinib group. Most of the patients (85.0%) were chemotherapy‐naïve. The mean duration of first‐line crizotinib treatment was 17.3 months in the brigatinib group and 22.7 months in the alectinib group. There were no significant differences in demographic characteristics between the brigatinib and alectinib groups, except for intracranial metastases, which tended to be more common in the alectinib group (41.2% vs. 69.2%, *p* = 0.058) (Table 1).

### 3.2 Treatment Efficacy

**FIGURE 1:**
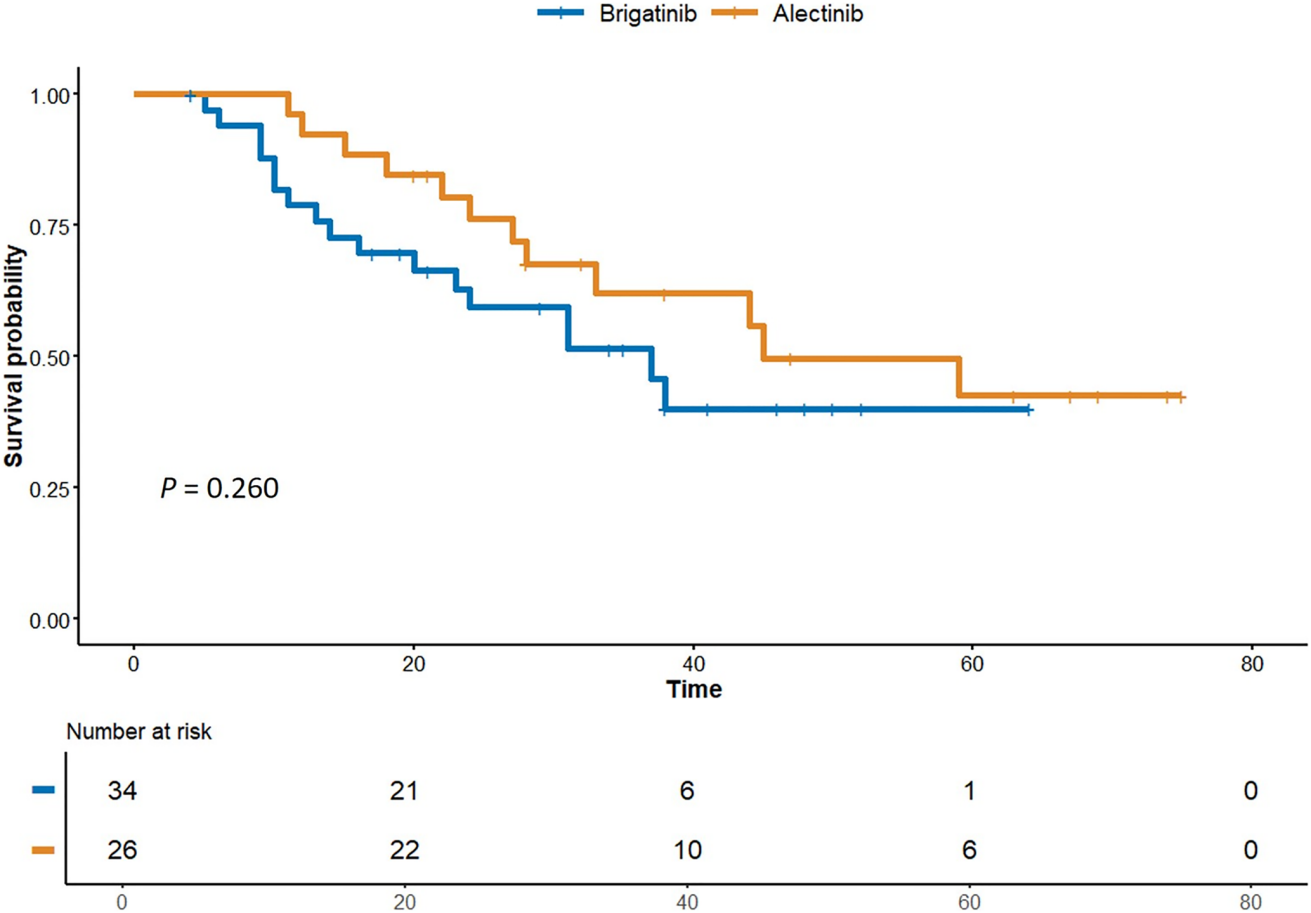
Comparison of progression‐free survival between brigatinib and alectinib in crizotinib‐refractory NSCLC patients.

At the date of primary data cutoff date (September 14, 2024), an event of disease progression or death occurred in 17 patients (50.0%) in the brigatinib group and 12 patients (46.2%) in the alectinib group. The 1‐, 2‐, and 3‐year PFS rates were 78.8%, 59.4%, and 51.5% in the brigatinib group and 92.3%, 76.2%, and 62.1% in the alectinib group, respectively. However, at 5 years, the PFS rates were comparable between the two groups (40.0% vs. 42.5%; log‐rank *p* = 0.260) (Figure 1).

**FIGURE 2:**
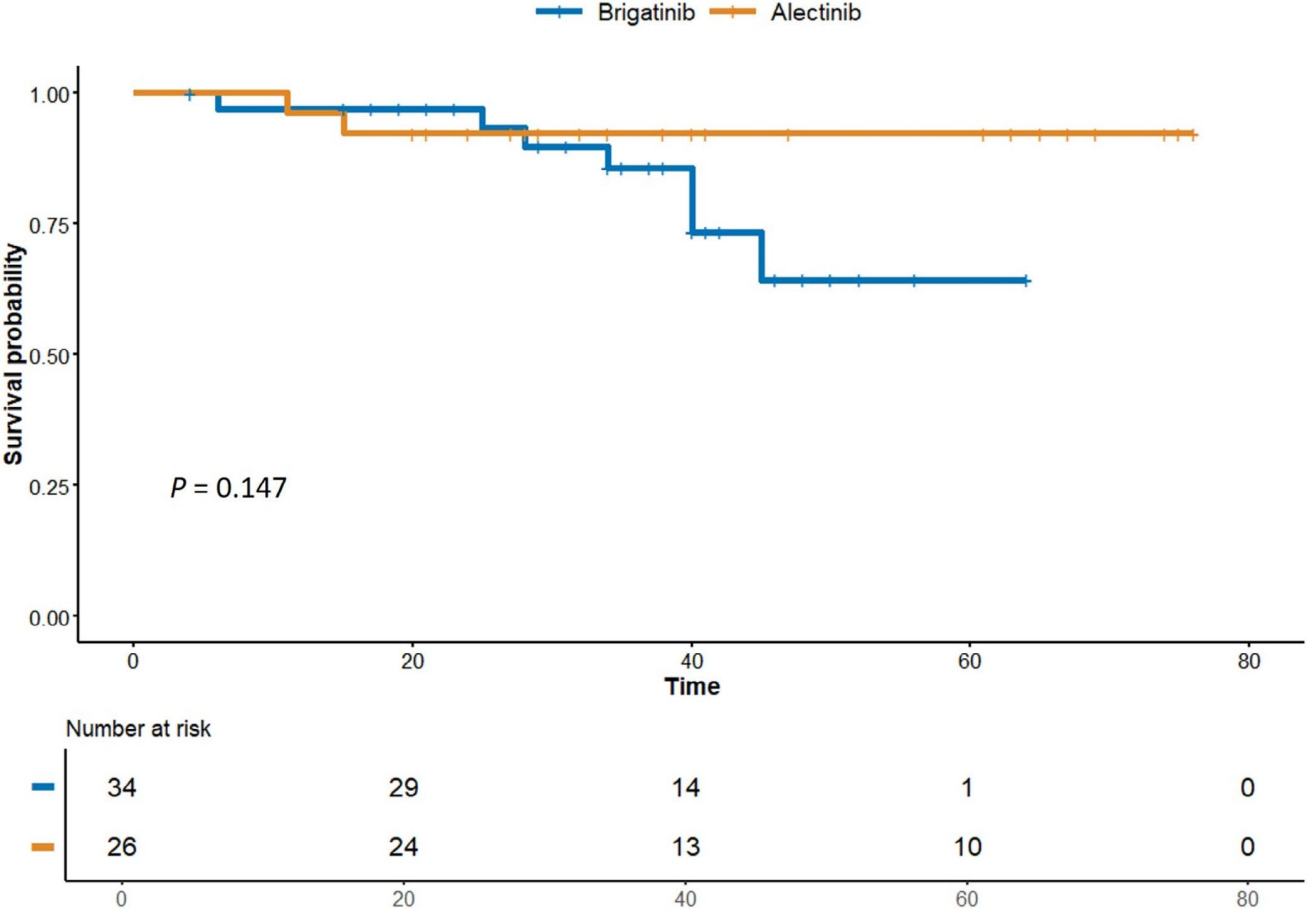
Comparison of overall survival between brigatinib and alectinib in crizotinib‐refractory NSCLC patients.

The ORR was 58.8% in the brigatinib group and 46.2% in the alectinib group, with no significant difference (*p* = 0.475) (Table 2). The median OS was not reached in either the brigatinib or the alectinib group, and there was no statistically significant difference between the two groups (*p* = 0.147) (Figure 2). The 24‐month OS rate was 97.0% in the brigatinib group and 92.3% in the alectinib group. The DCR was 100% in both the brigatinib and alectinib groups, respectively (*p* > 0.999) (Table 2).

**TABLE 2:** Comparison of best objective response rate between brigatinib and alectinib in crizotinib‐refractory NSCLC patients.

	Brigatinib	Alectinib	*p*
Number of patients	34	26	
Best response			0.475
Complete response	0 (0)	0 (0)	
Partial response	20 (58.8)	12 (46.2)	
Stable disease	14 (41.2)	14 (53.8)	
Progressive disease	0 (0)	0 (0)	
Objective response rate	19 (55.9)	12 (46.2)	0.627
Disease control rate	34 (100)	26 (100)	> 0.999

*Note:* Data are presented as number (%).

Abbreviation: NSCLC: non‐small cell lung cancer.

### 3.3 Intracranial Treatment Efficacy

**FIGURE 3:**
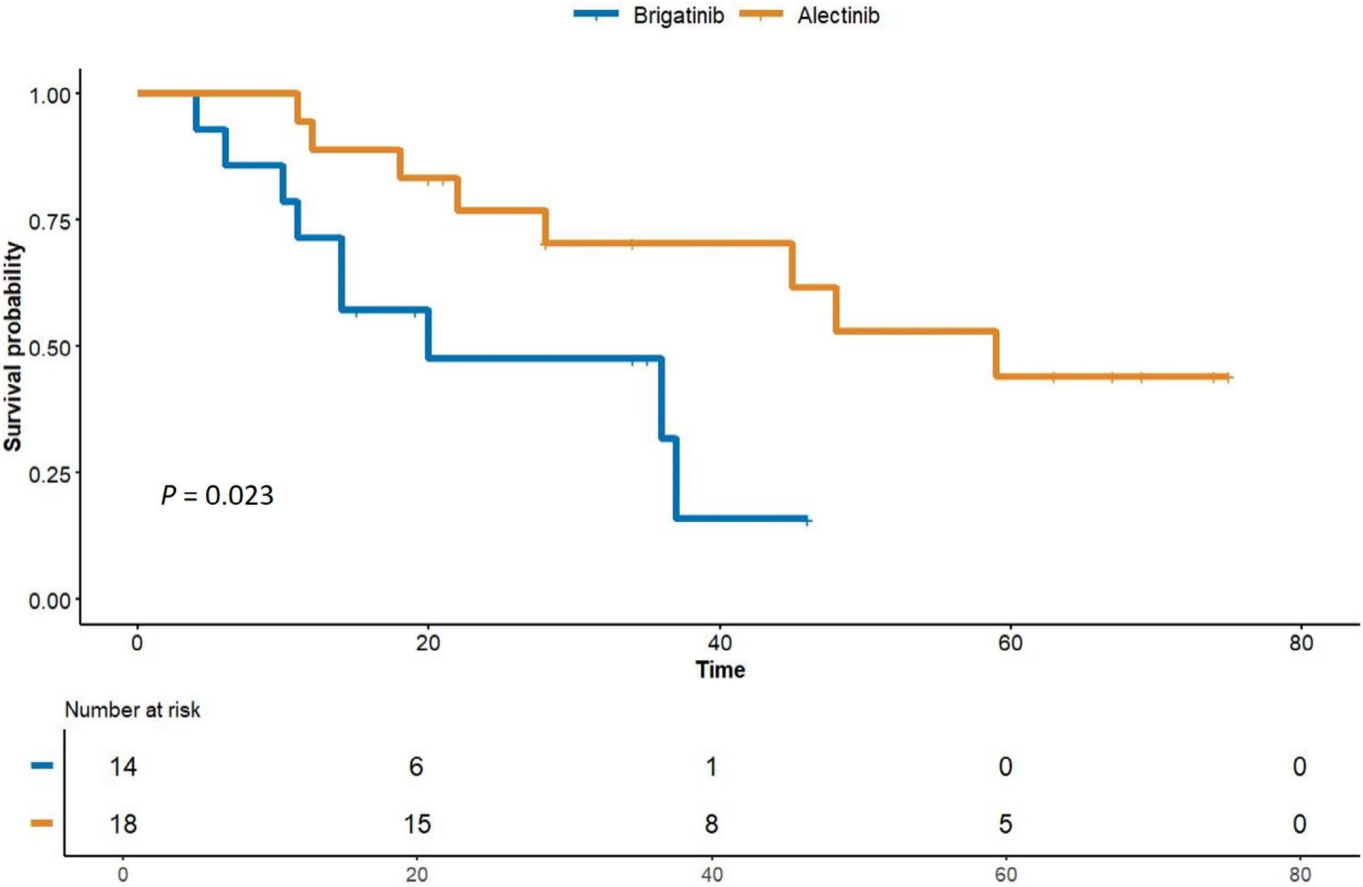
Comparison of intracranial progression‐free survival between brigatinib and alectinib in crizotinib‐refractory NSCLC patients.

The 1‐, 2‐, and 3‐year intracranial PFS rates were 71.4%, 47.6%, and 31.7% in the brigatinib group and 88.9%, 76.9%, and 70.5% in the alectinib group, respectively (log‐rank *p* = 0.023) (Figure 3). In the brigatinib group, no patients underwent surgical resection, 35.7% had received prior radiosurgery such as Gamma knife, and 21.4% had undergone whole‐brain radiotherapy (WBRT) before initiating second‐line ALK inhibitor treatment. In the alectinib group, 16.7% had undergone surgical resection, 55.6% received radiosurgery, and 5.6% were treated with WBRT (Table 1). The intracranial ORR was 64.3% for the brigatinib group and 94.4% for the alectinib group (*p* = 0.028) (Table 3).

**TABLE 3:** Comparison of intracranial best objective response rate between brigatinib and alectinib in crizotinib‐refractory NSCLC patients.

	Brigatinib	Alectinib	*p*
Number of patients	14	18	
Best response			0.088
Complete response	4 (28.6)	9 (50.0)	
Partial response	5 (35.7)	8 (44.4)	
Stable disease	5 (35.7)	1 (5.6)	
Progressive disease	0 (0)	0 (0)	
Objective response rate	9 (64.3)	17 (94.4)	0.028
Disease control rate	14 (100.0)	18 (100.0)	> 0.999

*Note:* Data are presented as number (%).

Abbreviation: NSCLC: non‐small cell lung cancer.

In the subgroup analysis of patients without prior local CNS therapy, the median intracranial PFS was 20 months with brigatinib and 59 months with alectinib (*p* = 0.1), and the intracranial ORR was 64.3% with brigatinib and 100% with alectinib, respectively (*p* = 0.028).

### 3.4 Safety and Tolerability

Table 4 shows adverse events leading to dose modification and treatment discontinuation during second‐line ALK inhibitor treatment. A total of 96.7% of patients experienced at least one adverse event (AE), with similar incidence in the brigatinib (97.1%) and alectinib (96.2%) groups. Grade ≥ 3 AEs occurred in 23.3% of all patients, with comparable rates in the brigatinib (20.6%) and alectinib (26.9%) groups. Detailed adverse events leading to grade ≥ 3 toxicity are shown in e‐Table S1. Among total grade ≥ 3 AEs, liver function test (LFT) elevation was the most common (8.3%), observed more frequently in the brigatinib group (11.8%) than in the alectinib group (3.8%). Pneumonitis leading to permanent treatment discontinuation occurred in 1 patient (2.9%) in the brigatinib group and in 1 patient (3.8%) in the alectinib group. One patient in the alectinib group experienced sudden death, with no obvious cause identified. Other grade ≥ 3 AEs included skin rash, acute kidney injury, seizure, and pleural effusion. Dose modifications due to AEs were more common in the brigatinib group than in the alectinib group (35.3% vs. 11.5%), whereas permanent discontinuations occurred more frequently in the alectinib group than in the brigatinib group (15.4% vs. 5.9%). The most common cause of permanent discontinuation was LFT elevation (6.7%), followed by pneumonitis (3.3%) (Table 4).

**TABLE 4:** Adverse events leading to dose modification and treatment discontinuation between brigatinib and alectinib in crizotinib‐refractory NSCLC patients.

	Total	Brigatinib	Alectinib
Number of patients	60	34	26
Any AEs	58 (96.7)	33 (97.1)	25 (96.2)
AEs ≥ grade 3	14 (23.3)	7 (20.6)	7 (26.9)
Dose modification	15 (25.0)	12 (35.3)	3 (11.5)
Temporary discontinuation	3 (5.0)	3 (8.8)	0 (0)
Permanent discontinuation	6 (10.0)	2 (5.9)	4 (15.4)
Pneumonitis	2 (3.3)	1 (2.9)	1 (3.8)
LFT elevation	4 (6.7)	1 (2.9)	3 (11.5)

Abbreviations: AE: adverse events; LFT: liver function test; NSCLC: non‐small cell lung cancer.

### 3.5 Subsequent Treatments

Regarding post‐study treatments, most patients received lorlatinib as third‐line therapy (88.0%), with comparable proportions in the brigatinib (84.2%) and alectinib (100.0%) groups. Cytotoxic chemotherapy was administered in 12.0% of patients, all in the brigatinib group. In the fourth‐line or beyond setting (*n* = 8), treatments included lorlatinib (25.0%), cytotoxic chemotherapy (62.5%), and the other regimen such as pembrolizumab (12.5%). Additionally, 17 patients underwent local CNS‐directed therapy, most commonly radiosurgery (94.1%), with one patient receiving whole‐brain radiotherapy (5.9%) (e‐Table 2).

## 4 Discussion

To the best of our knowledge, this is the first prospective real‐world study to compare the efficacy and safety of two second‐generation ALK tyrosine kinase inhibitors (TKIs) in ALK‐positive NSCLC patients who are refractory to first‐line crizotinib treatment. In our study, brigatinib showed a comparable PFS rate and ORR to alectinib in patients who experienced disease progression with first‐line crizotinib treatment, with a similar rate of adverse events.

In the past, crizotinib was the standard first‐line therapy for patients with advanced ALK‐positive NSCLC, demonstrating an ORR of 74% and a median PFS of about 11 months [9]. However, its efficacy is limited by the emergence of acquired resistance and a relatively short PFS, with the CNS being the most common site of disease progression [9]. Recent randomized clinical trials have demonstrated the efficacy of second‐generation TKIs, such as alectinib and ceritinib, as preferred first‐line treatments for patients with advanced ALK‐positive NSCLC [21, 22]. For example, in the ALEX trial, a randomized open‐label, phase‐3 trial, alectinib showed superior efficacy and lower toxicity compared to crizotinib in patients with ALK‐positive NSCLC [21]. Based on these data, alectinib has become the standard initial therapy for patients with ALK‐positive NSCLC.

Brigatinib is another second‐generation selective ALK inhibitor developed to overcome resistance to crizotinib. Subsequent clinical trials have shown that brigatinib is effective and well‐tolerated in both treatment‐naïve and crizotinib‐refractory ALK‐positive NSCLC patients [15, 18, 23, 24]. A randomized, open‐label, multicenter trial (the ALTA‐1 L trial) was conducted to compare the clinical efficacy of brigatinib and crizotinib in adult patients with advanced ALK‐positive NSCLC who had not been treated with ALK inhibitors. Camidge et al., in the ALTA‐1 L trial, reported a 3‐year PFS rate of 43% in the brigatinib group and 19% in the crizotinib group [23]. The median PFS was 24.0 months in the brigatinib group and 11.1 months in the crizotinib group (Hazard ratio [HR], 0.48; 95% CI, 0.35–0.66; *p* < 0.0001). During a median follow‐up period of 40.4 months for the brigatinib group and 15.2 months for the crizotinib group, ORR was 74% with the brigatinib group compared to 62% with the crizotinib group (Odds ratio [OR], 1.74; 95% CI [1.04–2.91]; *p* value = 0.033) [23]. Moreover, in the ALTA‐1 L trial, the confirmed intracranial objective response rate (ORR) in patients with measurable brain metastases at baseline was 78% in the brigatinib group and 26% in the crizotinib group (OR, 11.67; 95% CI, 2.15–63.27; *p* = 0.0101), suggesting robust intracranial efficacy [23].

However, there is a lack of data directly comparing the efficacy of brigatinib and alectinib in ALK‐positive NSCLC patients who experience disease progression after first‐line crizotinib treatment in the real‐world setting. Since brigatinib demonstrated potent activity against 17 ALK resistance mutations found in crizotinib, ceritinib, or alectinib [25], it was expected to exhibit superior efficacy compared to alectinib and to serve as a potential treatment option for patients refractory to alectinib. The phase 2J‐ALTA trial, a single‐arm, multicenter, open‐label study, evaluated the efficacy of brigatinib in Japanese patients with advanced ALK‐positive NSCLC who had progressed on alectinib [26]. Brigatinib demonstrated clinically meaningful efficacy, with an ORR of 34%, a DCR of 79%, and a median PFS of 7.3 months [26]. In contrast, in a multicenter, retrospective study involving 22 patients with advanced, alectinib‐refractory ALK‐positive NSCLC, brigatinib reported limited efficacy with a median PFS of 4.4 months and an ORR of 17% [27]. Notably, the open‐label, randomized, multicenter, international, phase 3 trial (ALTA‐3) recently conducted a head‐to‐head comparison of the efficacy and safety of brigatinib versus alectinib in ALK‐positive NSCLC who experienced disease progression after crizotinib treatment [19]. The median PFS, as assessed by the blinded independent review committee (BIRC), was 19.3 months for the brigatinib group and 19.2 months for the alectinib group (HR, 0.97; 95% CI [0.66–1.42], log‐rank *p* = 0.867). In addition, in patients presenting with measurable brain metastases at baseline, BIRC‐assessed confirmed intracranial ORR was 73% with brigatinib and 68% with alectinib, suggesting comparable clinical efficacy between brigatinib and alectinib [19]. However, questions remain regarding the comparative clinical efficacy of the two agents in patients with crizotinib‐refractory ALK‐positive NSCLC, particularly in real‐world settings beyond the context of highly controlled clinical trials, especially among Asian patients.

In this real‐world, multicenter, prospective cohort study, there were no significant differences in clinical outcomes between brigatinib and alectinib in patients with ALK‐positive NSCLC refractory to crizotinib, aligning with previous studies [19, 28]. Although a direct comparison is limited by differences in study design, patient characteristics, and follow‐up duration, the 3‐year PFS rates observed in this study (51.5% for brigatinib and 62.1% for alectinib) appear numerically favorable compared with the ALTA3 trial, which reported median PFS of 19.3 months for brigatinib and 19.2 months for alectinib (no PFS rates reported) [19]. This may be explained by differences in prior chemotherapy history: over 80% of patients in this study were treatment‐naïve prior to crizotinib, whereas approximately 30% of patients in the aforementioned study had received more than one prior line of chemotherapy [19]. In addition, the proportion of patients with baseline brain metastases was lower in our study (53.3%), particularly in the brigatinib group (41.2%), compared to approximately 60% reported in the previous study [19]. Moreover, the duration of prior crizotinib treatment was notably longer in our study (mean duration, 17.3 months in the brigatinib and 22.7 months in the alectinib), compared to the previous study (median duration, 16.0 months and 16.8 months, respectively) [19], suggesting that the tumors in our cohort may have been more sensitive to ALK inhibitors. Similarly, the numerically lower PFS rates observed in the brigatinib group compared to the alectinib group in this study may also be attributable to the same reason.

In our study, the intracranial PFS rates and ORR were higher in the alectinib group compared to the brigatinib group. The intracranial ORR in the brigatinib group was comparable to that reported in the ALTA‐3 trial (73%), whereas the ORR in the alectinib group was notably higher than that observed in the same trial (68%) [19]. This discrepancy may be partly explained by the difference in prior local treatments for brain metastases. In the ALTA‐3 trial, only 23% of patients in the alectinib group had received brain radiotherapy (radiosurgery and WBRT), whereas approximately 60% of patients in the brigatinib group in our study had undergone local treatments. In addition, a higher proportion of patients in the brigatinib group had received prior WBRT compared with the alectinib group (21.4% vs. 5.6%), suggesting a greater baseline CNS disease burden. As WBRT is typically considered for multiple or widespread brain metastases, this baseline difference may have contributed to the relatively inferior intracranial efficacy with brigatinib. However, in our subgroup analysis limited to patients without prior local CNS therapy (7 patients per group), alectinib still demonstrated a longer median intracranial PFS than brigatinib (59 vs. 20 months), although these results should be interpreted with caution given the small sample size. Taken together, these findings suggest that, while prior CNS‐directed interventions may partly account for the observed differences, intrinsic differences in intracranial activity between the two agents are also likely relevant. Our findings suggest that both brigatinib and alectinib are generally well tolerated, with a safety profile consistent with those reported in previous studies [15, 18, 21, 24], and no new safety concerns were observed. In our study, pneumonitis leading to permanent discontinuation occurred in one patient in both the brigatinib and alectinib groups, with incidences of 2.9% and 3.8%, respectively, comparable to that of the ALTA trial (6%) [18]. As pneumonitis has been reported to occur within the first week of brigatinib treatment, patients receiving brigatinib should be closely monitored for the development or worsening of respiratory symptoms, especially during the first week of treatment [18, 29]. Notably, LFT elevation was the most common cause of permanent treatment discontinuation in the overall population, and LFT elevation leading to discontinuation occurred more frequently in the alectinib group in our study, in line with the previous study [30]. In the updated safety analysis of the ALEX study [30], which included patients treated with alectinib or crizotinib, elevations in ALT and AST were reported in 17.1% and 15.8% of patients, respectively, with grade 3–5 elevations occurring in 4.6% and 5.3%. These findings highlight the importance of routine LFT monitoring, especially in patients with baseline hepatic impairment receiving alectinib. Taken together, alectinib can be considered for patients at higher risk of drug‐induced pneumonitis, such as those with interstitial lung disease, while brigatinib may be a more appropriate choice for patients with liver dysfunction or elevated muscle enzymes.

This study has several limitations. First, as the study population consisted of Korean patients, the generalizability of our findings may be limited, and the relatively small sample size may have limited the statistical power. However, baseline characteristics of this population were similar to those in previous studies [19, 28]. Second, both treatment responses and adverse events were assessed by the investigators, which may have introduced observer bias. Third, although this was a prospective study, the intracranial disease status at the initiation of second‐line ALK inhibitor therapy, particularly whether intracranial lesions were active or stable, was not assessed. This limitation may affect the accuracy of intracranial ORR assessment, highlighting the need for further studies to better define the intracranial efficacy of second‐line ALK inhibitors following crizotinib failure. Fourth, since this was a real‐world study rather than a randomized controlled trial, treatment selection was based on routine clinical practice, and therefore not all potential confounding factors could be fully adjusted for. This may have introduced selection bias, and the observed treatment effect should be interpreted with caution in light of this limitation. To elucidate the relative impact of alectinib and brigatinib after crizotinib failure, further randomized or prospective studies are needed. Finally, ALK fusion was confirmed only by FISH or IHC, which, despite being standard methods during the study period, may carry a risk of false positivity. More advanced assays such as NGS or ctDNA were not available for most patients.

In conclusion, systemic efficacy was not significantly different between brigatinib and alectinib in crizotinib‐refractory ALK‐positive NSCLC; however, alectinib was associated with more favorable intracranial PFS and ORR, which may be partly explained by differences in prior brain‐directed local treatments. Further studies with larger sample sizes are needed to validate these findings.

## Author Contributions

Conceptualization: Chang Min Choi. Methodology: Min Jee Kim, Chang Min Choi. Formal analysis: Min Jee Kim. Data curation: Hyun Seok Kwak, Eun Nim Koh, Cheol‐Kyu Park, Young‐Chul Kim, In‐Jae Oh, Seung Joon Kim, Jun Hyeok Lim, Jeong‐Seon Ryu, Chang Min Choi. Writing original draft: Min Jee Kim. Writing – review and editing: In‐Jae Oh, Chang Min Choi. Visualization: Min Jee Kim. Funding acquisition: Chang Min Choi.

## Consent

All patients provided written informed consent before screening.

## Conflicts of Interest

The authors declare no conflicts of interest.

## Supporting information


**Data S1:** tca70175‐sup‐0001‐Supinfo.docx.

## Data Availability

The data that support the findings of this study are available from the corresponding author upon reasonable request.
