# Supplementary material for: Real‐World Outcomes of Brigatinib Compared to Alectinib as a Second‐Line Therapy After Crizotinib in Advanced Anaplastic Lymphoma Kinase Positive Non‐Small Cell Lung Cancer Patients
Source: Thorac Cancer. 2025 Oct 10;16(19):e70175. doi: 10.1111/1759-7714.70175 (PMC12514014; doi:10.1111/1759-7714.70175)
Supplement: Supplementary file 1 — Data S1: tca70175‐sup‐0001‐Supinfo.docx. [file TCA-16-e70175-s001.docx]

**Supplementary file**

**Real-world outcomes of brigatinib compared to alectinib as a second-line therapy after crizotinib in advanced anaplastic lymphoma kinase positive non-small cell lung cancer patients**

Min Jee Kim^1^, Hyun Seok Kwak^1^, Eun Nim Koh^1^, Cheol-Kyu Park^2^, Young-Chul Kim^2^, In-Jae Oh^2^, Seung Joon Kim^3^, Jun Hyeok Lim^4^, Jeong-Seon Ryu^4^, Chang Min Choi^1*^

^1^Department of Pulmonary and Critical Care Medicine, Asan Medical Center, University of Ulsan College of Medicine, Seoul, Korea

^2^Department of Internal Medicine, Chonnam National University Medical School, Hwasun, Jeonnam, 58128

^3^Division of Pulmonology, Department of Internal Medicine, Seoul St. Mary’s Hospital, College of Medicine, The Catholic University of Korea, Seoul, Republic of Korea

^4^Division of Pulmonology, Department of Internal Medicine, Inha University Hospital, Inha University College of Medicine, Incheon, Republic of Korea

***Correspondence:**

Chang Min Choi, MD, PhD

Department of Pulmonary and Critical Care Medicine, University of Ulsan College of Medicine, Asan Medical Center, 88 Olympic-ro 43-gil, Songpa-gu, Seoul, Republic of Korea

Tel.: 82-2-3010-5902

Fax: 82-2-3010-6968

E-mail: ccm@amc.seoul.kr

e-Table 1. Detailed adverse events leading to Grade 3 or higher toxicity.

|  | Total | Brigatinib | Alectinib |
| --- | --- | --- | --- |
| Number of patients | 60 | 34 | 26 |
| LFT elevation | 5 (8.3) | 4 (11.8) | 1 (3.8) |
| Pneumonitis | 4 (6.7) | 2 (5.9) | 2 (7.7) |
| Skin rash | 1 (1.7) | 0 (0) | 1 (3.8) |
| Sudden death | 1 (1.7) | 0 (0) | 1 (3.8) |
| Acute kidney injury | 1 (1.7) | 1 (2.9) | 0 (0) |
| Seizure | 1 (1.7) | 0 (0) | 1 (3.8) |
| Pleural effusion | 1 (1.7) | 0 (0) | 1 (3.8) |

NSCLC: non-small cell lung cancer, AEs: adverse events, LFT: liver function test

e-Table 2. Subsequent treatments after brigatinib or alectinib treatment in patients with ALK positive NSCLC.

|  | Total | Brigatinib | Alectinib |
| --- | --- | --- | --- |
| 3^rd^ line |  |  |  |
| Number of patients | 25 | 19 | 6 |
| Loratinib | 22 (88.0) | 16 (84.2) | 6 (100.0) |
| Cytotoxic therapy | 3 (12.0) | 3 (15.8) |  |
| 4^th^ line or beyond |  |  |  |
| Number of patients | 8 | 7 | 1 |
| Loratinib | 2 (25.0) | 2 (28.6) | 0 (0) |
| Cytotoxic therapy | 5 (62.5) | 4 (57.1) | 1 (100.0) |
| Other^*^ | 1 (12.5) | 1 (14.3) | 0 (0) |
| Local CNS therapy |  |  |  |
| Number of patients | 17 | 10 | 7 |
| Radiosurgery | 16 (94.1) | 10 (100.0) | 6 (85.7) |
| WBRT | 1 (5.9) | 0 (0) | 1 (14.3) |

ALK: anaplastic lymphoma kinase, NSCLC: non-small cell lung cancer, CNS: central nervous system, WBRT: whole brain radiothreapy

^*^Other: includes pembrolizumab
